# Complete remission of pancreatic head desmoid tumor treated by COX-2 inhibitor—a case report

**DOI:** 10.1186/s12957-016-0944-z

**Published:** 2016-07-22

**Authors:** Yu-Chieh Wang, Jia-Uei Wong

**Affiliations:** Division of General Surgery, Department of Surgery, Cathay General Hospital, Taipei, Taiwan

**Keywords:** Desmoid tumor, Aggressive fibromatosis, Pancreatic head, Non-steroidal anti-inflammatory drugs, Cyclooxygenase-2 inhibitor

## Abstract

**Background:**

Desmoid tumors (DTs) are non-metastatic, locally aggressive neoplasms with high postoperative recurrence rates. The pancreas is an extremely rare location for DTs. The local control of DTs is challenging. Surgery and radiotherapy are currently the principal treatment modalities for DTs; however, some resections might not be radical, and radiotherapy has several drawbacks. Therefore, many studies have been focusing on the molecular pathways involved in DTs in order to develop molecular-targeted therapies or chemotherapy. Cyclooxygenase-2 (COX-2) has been demonstrated to play a role in the growth of DTs, and the pharmacologic blockade of COX resulted in decreased cell proliferation in desmoid cell cultures in vitro.

**Case presentation:**

Herein, we report a 57-year-old woman who presented with recurrent epigastric pain and weight loss. An abdominal computed tomography scan showed an approximately 10-cm mass over the pancreatic head region and dilatation of the pancreatic duct. Tumor biopsy and bypass surgery were performed. A DT was confirmed on pathologic analysis. After resection, we prescribed treatment with the COX-2 inhibitor celecoxib. The patient showed complete remission and there was no local recurrence or distant metastasis within the 24-month follow-up period.

**Conclusions:**

The outcome of this case study is encouraging, and long-term follow-up studies are required to establish the effect of treatment with celecoxib on the prognosis of DTs.

## Background

A desmoid tumor (DT), also known as aggressive fibromatosis, is a rare soft tissue neoplasm. Patients with a history of familial adenomatous polyposis, surgery, or pregnancy show higher incidence rates of DT. Although this type of tumor has a benign histology, it is locally invasive. Intra-abdominal DT has a low incidence rate, and DTs of pancreatic origin are rare. The treatment of these rare tumors is challenging because of their potential for recurrence.

Resection with negative surgical margins is the most successful treatment modality for DTs [1]. For patients who refuse surgery or are not surgical candidates, radiotherapy and medical therapy might be considered.

Herein, we report a 57-year-old woman who developed DT at a rare location. A cystic and solid mixed lesion was noted at the pancreatic head, and the final pathologic analysis indicated DT. The patient received only medical therapy after the operation and achieved complete remission.

## Case presentation

**Fig. 1:**
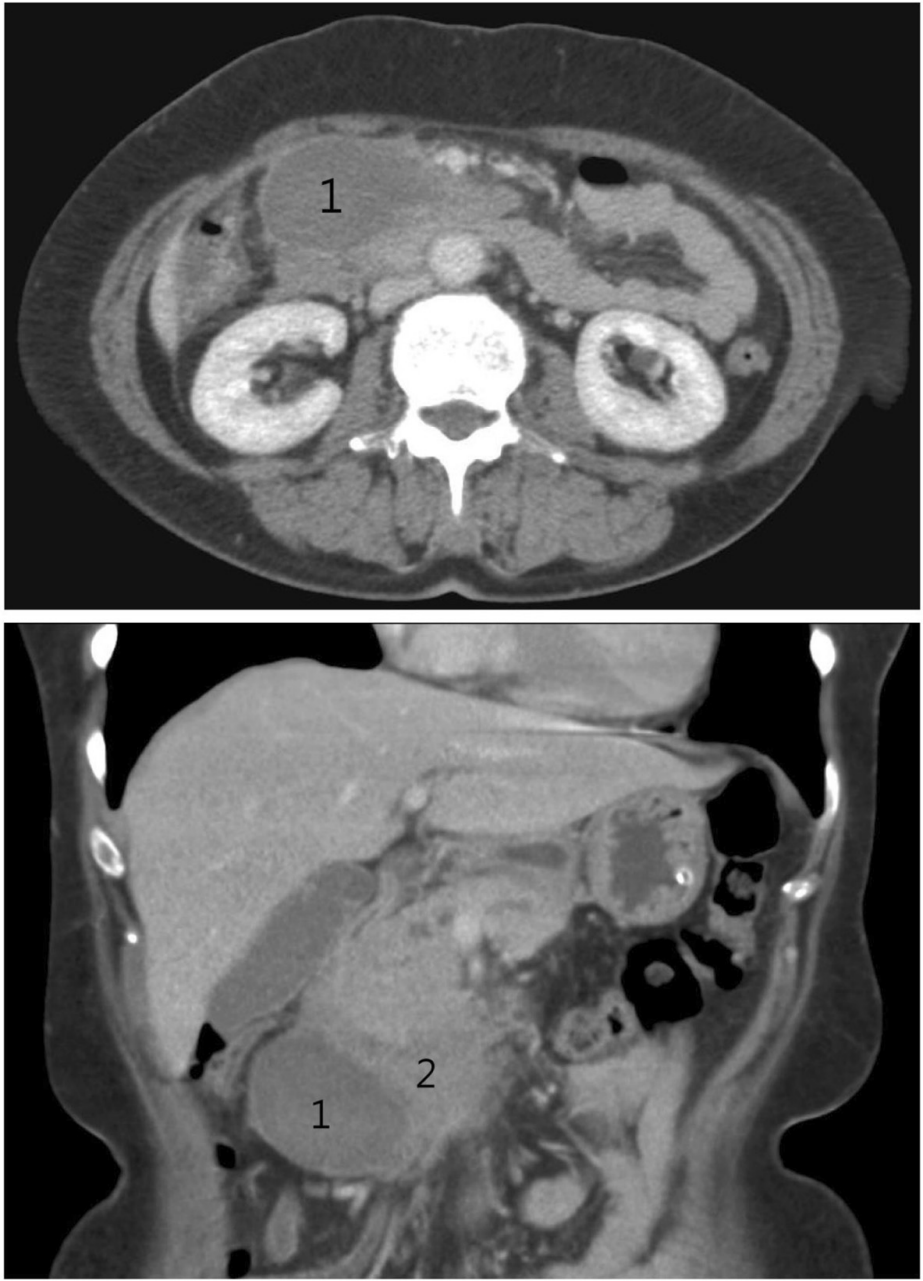
Cystic part (1) and solid part (2) of the pancreatic head tumor

A 57-year-old woman had underlying systemic iron deficiency anemia. She presented to our outpatient clinic with abdominal pain in March 2013. Panendoscopy and colonoscopy were performed and no organic lesions were detected in the upper and lower GI tract, and only gastritis was noted. However, the abdominal discomfort could not be eliminated and it worsened. Because of progressive abdominal pain, the patient was transferred to the emergence department after 1 week. Abdominal computed tomography (CT) (Fig. 1) revealed a marked, approximately 10-cm mass in the pancreatic head. The pancreatic head tumor was initially diagnosed and she was then admitted for further examination.

During hospitalization, laboratory analysis did not show hyperbilirubinemia and elevated amylase or lipase levels. The levels of tumor markers including carcinoembryonic antigen, carbohydrate antigen 19-9, and carbohydrate antigen 125 were within normal limits. CT-guided aspiration of the cystic component was performed, and cytological analysis showed only some macrophages. We performed surgical exploration on March 22, 2013.

**Fig. 2:**
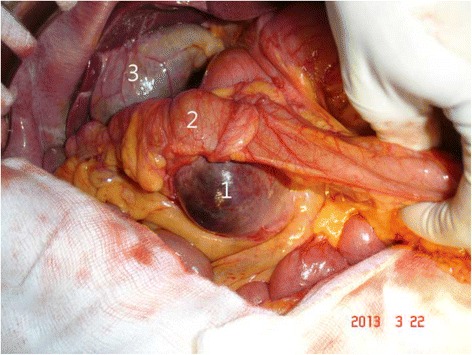
The tumor (1) was noted from the pancreas and T-colon (2) was just above the tumor with mesocolon compressed. Although the mass effect of tumor made the patient abdominal pain, bile duct was not compressed to cause obstructive jaundice and gallbladder (3) was not distended

**Fig. 3:**
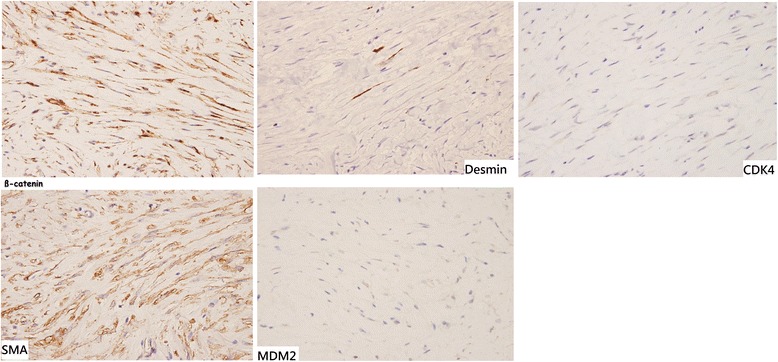
Pathology showed positive staining for SMA, desmin, and beta-catenin. Desmoid tumor was confirmed in final diagnosis

During the operation, we found that the lesion was approximately 10 cm in size with cystic and solid components (Fig. 2). The tumor appeared to originate from the pancreatic head and presented as a cystic hematoma protruding downward into the mesocolon. We performed partial cystectomy for hematoma evacuation, and tumor tissue was obtained from the pancreatic head for pathologic analysis. Examination of frozen tissue section did not indicate any malignancy. Finally, gastrojejunostomy bypass was performed without radical resection of the tumor. The patient showed good postoperative recovery. The final pathologic analysis confirmed the DT diagnosis and immunohistochemical staining showed focal positivity for smooth muscle actin and desmin but negativity for Mdm2, CDK4, and CD34. The tumor showed strong positivity for beta-catenin (Fig. 3).

**Fig. 4:**
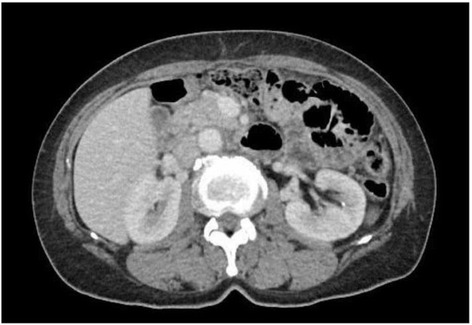
Follow-up CT in 18 months later showed marked regression of pancreatic desmoid tumor under celecoxib treatment

The patient received one 200-mg tablet daily of the non-steroidal anti-inflammatory drug (NSAID) celecoxib for half a year. We obtained a follow-up abdominal CT scan in October 2013 after 6 months of celecoxib treatment. The CT images showed a marked regression of the existing pancreatic head mass and complete disappearance of the cystic lesion. Therefore, the dose was modified from one tablet daily to one tablet every other day with the same dose of celecoxib starting from the date of the 6-month follow-up until now. The abdominal CT scan obtained at the 18-month follow-up in September 2014 showed no local recurrence (Fig. 4). There is no side effect noted by the patient in the period of celecoxib treatment. The patient is still receiving celecoxib treatment for the control DT.

### Discussion

DT originates from the abnormal proliferation of myofibroblasts. It is a very rare disease and presents with a benign pathology; however, it exhibits local invasiveness indicating clinical malignancy. The DT location can be classified as extra-abdominal, abdominal, or intra-abdominal. Genetic screening for defects showed that mutations in the FAP gene were a risk factor that was more frequently associated with intra-abdominal DT [2]. Other studies showed that intra-abdominal DT frequently had pelvic and mesenteric origins. DTs originating from pancreas are very rare. To date, only 12 cases of pancreatic DT have been reported [3–13]. Among these articles, only three of them reported DTs that originated in the pancreatic head, and all patients underwent tumor resection [11–13]. The present case is the fourth case of DT originating from the pancreatic head; however, it is the only case treated by medication and achieved completed remission.

The clinical presentation of most patients with DT is usually asymptomatic and otherwise presents non-specific abdominal pain. DT diagnosis could not be confirmed via laboratory analysis or radiological images. Therefore, the definitive diagnosis of DT could only be based on histological and immunohistochemical findings [14]. In present case, DT diagnosis was confirmed by the final immunohistochemistry staining that showed strong positivity for beta-catenin and focal positivity for smooth muscle actin and desmin.

The current first-line treatment for DT is complete resection with free margins. Sometimes, performing resection with wide, free margins for the prevention of tumor recurrence is difficult because of the locally invasive behavior of the tumor. Chemotherapy, molecular-targeted therapy, and radiotherapy are considered in cases with high surgical risk. Another case that was successfully treated with NSAIDs was previously reported [15].

Further analysis showed the stabilization of the beta-catenin protein in DT caused by mutation in either the APC or beta-catenin gene [16]. If mutated, beta-catenin accumulates in the cell and activates the T cell factor, which triggers the transcription of target genes including COX-2 [17]. Therefore, an elevated COX-2 protein level was observed in DT; COX-2 partially regulates proliferation through beta-catenin stabilization [18]. Therefore, the degradation of this protein or reduction of the nuclear accumulation of beta-catenin might decrease tumor growth. A molecular model suggests an interaction between NSAIDs and beta-catenin. However, the mechanism of beta-catenin inhibition by NSAIDs has not yet been elucidated.

In the present case, the patient achieved complete remission on treatment with a COX-2 inhibitor, celecoxib. The initial plan was to treat the patient with tamoxifen; however, owing to its side effect, we did not proceed with this treatment option. At the end of the 2-year follow-up, the patient showed no tumor recurrence with maintenance therapy using celecoxib.

Tanaka et al. [15] reported that a patient who received etodolac, another COX-2 inhibitor, only achieved partial remission. NSAIDs indeed have a cytoreductive effect on DTs. However, different types of NSAIDs appear to have different efficacies in DT size reduction.

Although resection is the only radical treatment option for DT according to the current consensus, medical therapy also has an important role in the treatment of patients with comorbidities or in those where resection is contraindicated. Chemotherapy has a relatively high complication rate for patients, and the results of radiotherapy treatment were not optimistic. Therefore, NSAIDs offer another treatment option for DT with fewer side effects. However, additional clinical trials and molecular analyses should be performed to test the effect of NSAIDs in the treatment of DTs.

## Conclusions

Complete resection was reported as the first-line treatment choice in the reviewed case reports. However, in this case, single medical therapy using COX-2 inhibitor had excellent treatment effects on sporadic and non-FAP-associated DT. The details of the mechanisms should further be investigated and the efficacies of different NSAIDs should be established.

## Abbreviations

COX-2, cyclooxygenase-2; DT(s), desmoid tumor(s); NSAID, non-steroidal anti-inflammatory drug
